# Considering a neuropsychiatric obsessive–compulsive phenotype

**DOI:** 10.1192/j.eurpsy.2023.1964

**Published:** 2023-07-19

**Authors:** M. Pinho, D. O. Martins, P. S. Martins, L. Gomes, S. Carvalho

**Affiliations:** Hospital de Magalhães Lemos, Porto, Portugal

## Abstract

**Introduction:**

Up to 30% of individuals with obsessive-compulsive disorder (OCD) present with a current or past history of tics. Simultaneously, OCD is one of the most frequent psychiatric comorbidities in patients with primary tic disorders (TD), such as Tourette syndrome.

**Objectives:**

We present a literature review about the relationship between OCD and movement disorders, including its potential implications.

**Methods:**

A literature review is performed on PUBMED, using the next keywords: "obsessive-compulsive disorder”, “comorbidity”, “movement disorders” and “tic disorders” We focused on data from systematic reviews, clinical trials and meta-analysis published in English on last 10 years.

**Results:**

Goal-directed behaviour, such as compulsions, is orchestrated by the basal ganglia, through parallel but interconnected frontal–striatal circuits. Dysfunction of these circuits is known to play a role in the pathogenesis of TD and may also underlie OCD.

The most common movement disorders comorbid with obsessive-compulsive disorder (OCD) are indeed TD, with obsessive-compulsive symptoms (OCS) occurring in up to 90% of Tourette syndrome cases. OCD comorbid with TD associates with higher frequencies of hoarding, counting rituals, intrusive violent and sexual thoughts/images, somatic obsessions and repetitive movement compulsions. It also associates with earlier age of onset, higher frequency of sensory phenomena, higher male prevalence and familial aggregation.

However, OCD and OCS are also highly prevalent in choreatic movement disorders, such as Huntington’s disease and rheumatic fever with Sydenham’s chorea. There is also evidence for a correlation between streptococcal infections, autoimmunity, tic disorders and OCD, as seen in Pediatric Autoimmune Neuropsychiatric Disorders Associated with Streptococcal infections (PANDAS).

**Conclusions:**

Current evidence shows OCD and movement disorders may share dysfunctional brain circuits, resulting in a neuropsychiatric obsessive–compulsive phenotype, which may differ in terms of clinical characteristics and management.

**Disclosure of Interest:**

None Declared

